# Dietary Isorhamnetin Intake Is Inversely Associated with Coronary Artery Disease Occurrence in Polish Adults

**DOI:** 10.3390/ijerph191912546

**Published:** 2022-10-01

**Authors:** Joanna Popiolek-Kalisz, Emilia Fornal

**Affiliations:** 1 Clinical Dietetics Unit, Department of Bioanalytics, Medical University of Lublin, ul. Chodzki 7, 20-090 Lublin, Poland; 2 Department of Cardiology, Cardinal Wyszynski Hospital in Lublin, al. Krasnicka 100, 20-718 Lublin, Poland; 3 Department of Bioanalytics, Medical University of Lublin, ul. Jaczewskiego 8b, 20-090 Lublin, Poland

**Keywords:** flavonols, food frequency questionnaire, quercetin, cardiovascular disease, isorhamnetin

## Abstract

The role of antioxidative agents in coronary artery disease (CAD) has been investigated, but the analysis of specific flavonols intake in Polish adults requires validated tools. The aim of this study was to estimate the dietary intake of flavonols in CAD patients by creating a food frequency questionnaire (FFQ) dedicated for this purpose in Polish adults. The FFQ included 140 products from 12 food groups. The study involved 103 adult respondents (43 CAD patients and 60 healthy controls). Mean daily intakes of total flavonols, quercetin, kaempferol, myricetin and isorhamnetin were calculated as absolute values and quartiles. Mean daily intakes of 12 main food categories and 27 subcategories were calculated as portions and quartiles. The validity test revealed high correlation for total flavonols, kaempferol, myricetin and isorhamnetin and moderate for quercetin. In the reproducibility analysis, the correlation was high for total flavonols, quercetin, kaempferol and myricetin, moderate for isorhamnetin and high for all 12 categories and 25 out of 27 subcategories of the tested food groups. The application of the FFQ in healthy adults and CAD patients revealed that dietary intakes of total flavonols and proportional intakes of kaempferol and isorhamnetin in Polish adults and CAD patients are higher than in most other European countries, while the proportional intakes of quercetin and myricetin are lower than in most European countries. The comparison between CAD patients and the healthy controls revealed significant differences in dietary isorhamnetin intake (*p* = 0.002). The results suggest that dietary isorhamnetin could have a potential role in CAD prevention.

## 1. Introduction

Nutritional epidemiology plays an important role in the studies focusing on modifiable risk factors of different diseases [1,2]. Dietary patterns are related to many chronic diseases, including obesity, diabetes and cardiovascular diseases. The development of chronic diseases is usually related to long-term dietary habits. However, prolonged observation requires constant long-lasting dietary log reporting or retrospective analysis. In dietetics studies, food frequency questionnaires (FFQs) are used for the assessment of frequency of consumption and qualitative data evaluation, due to their relatively low cost, time effectiveness and ability to measure usual consumption in large populations. However, in recent years, there has been an increasing number of studies showing the use of new FFQs for the consumption of specific nutrients [3]. Additionally, numerous studies investigating the relationships between the dietary intakes of selected agents and health outcomes are based on food frequency questionnaires [4,5], but many of the questionnaires used are not validated.

The connections between noncommunicable chronic diseases and nutrition have already been established; however, the directed links between selected compound intakes and different diseases’ incidence or progression are still under investigation. The role of specific antioxidative agents in noncommunicable chronic disease prevention including coronary artery disease (CAD) has been an area of interest for many years [6,7,8,9]. Flavonols are a group of flavonoids differentiated by their chemical structure including 3-hydroxyflavone backbone. The most abundant individual flavonol is quercetin, followed by kaempferol, myricetin and isorhamnetin; however, this group includes also morin, galangin, fisetin, kaempferide, azaleatin, natsudaidain, pachypodol and rhamnazin, which are less prevalent [10,11,12,13].

Flavonols are present mainly in fruits, vegetables and tea. The major dietary contributors to everyday flavonol intake in the US are onions, tea and apples [14]. Other flavonol-rich products are: kale, lettuce, tomatoes, broccoli, grapes, berries and red wine [10,13]. They are known for their antioxidant properties, and thus, flavonols intake could have a role in the modification of cancer risk, metabolic syndrome or cardiovascular risk [15,16,17,18,19]. Some tools for general polyphenol intake assessment have been validated, but, as mentioned above, there has not been created any validated tool dedicated to specific flavonol intake assessment, which would be promising in terms of the future investigation of links between specific compound intakes and chronic diseases development [20].

As mentioned above, the role of antioxidants, including flavonols, in cardiovascular protection has been deeply investigated [17,19]. There are suggestions that specific flavonols’ supplementation could be beneficial in CAD prevention; however, there are not many studies linking habitual specific dietary flavonol intake and CAD development [19]. Moreover, the studies highlighted general differences in dietary intake of flavonols in American and various European populations, but none of the studies included Polish populations [13,21]. The aim of this study was to assess the dietary intake of flavonols in CAD patients by creating and validating a food frequency questionnaire for total and selected (quercetin, kaempferol, myricetin, isorhamnetin) flavonols intake in Polish adults.

## 2. Materials and Methods

**Figure 1:**
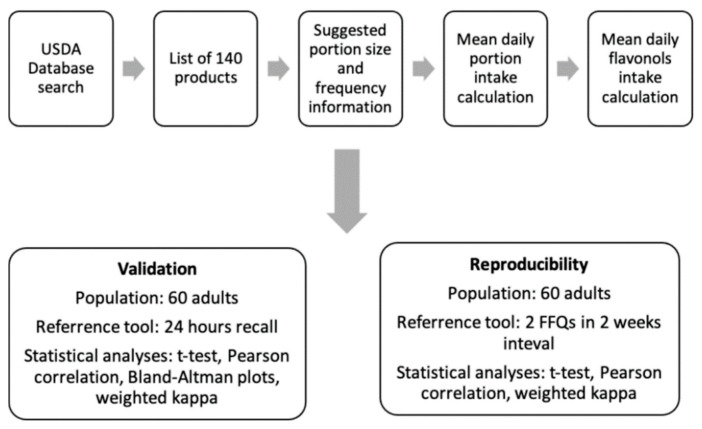
The scheme of the FFQ preparation and validation steps.

The study was based on two major parts, the FFQ preparation and validation in Polish adults and then the assessment of flavonol intake in CAD patients with the validated FFQ. The FFQ preparation was divided into three major steps presented in Figure 1: general preparation of the FFQ, validation and FFQ reproducibility control. The steps are described below.

### 2.1. Preparation of the Food Frequency Questionnaire

The USDA Database for Flavonoid Content of Selected Foods (the latest release, 3.2) was searched for products containing quercetin, kaempferol, isorhamnetin or myricetin [22]. A list of 140 products was created. The products were organized into main categories: vegetables, legumes, fruits, herbs and spices, nuts and seeds, jam and honey, wholegrain products, cocoa, tea and coffee, other nonalcoholic beverages (juices) and alcoholic beverages, and 27 subcategories [Supplementary Material File S1]. The suggested portions were introduced on the basis of typical servings in everyday life (e.g., one piece, a glass). The amount of a suggested portion was described by both a descriptor serving (e.g., a piece, a glass) and a weight in grams.

The question asked in the FFQ was: “How often during the last year did you consume the suggested portion of the following product?” The respondents could choose between the following options: never or almost never, once a month, few times a month (please give a number of times per month), once a week, few times a week (please give a number of times per week), once a day, few times every day (please give a number of times per day) [Supplementary Material File S2]. Then, on the basis of the declared frequency of selected product consumption, the mean daily consumption of each product was calculated.

The amounts of quercetin, kaempferol, isorhamnetin and myricetin in each suggested portion were based on the data available in the USDA database. Finally, the mean daily intakes of quercetin, kaempferol, isorhamnetin and myricetin were calculated. Total flavonol intake was calculated by adding the values for quercetin, kaempferol, isorhamnetin and myricetin.

The 24 h recall was conducted collaterally in the same participants. The respondents were asked to describe in detail all the products they had eaten on the previous day and their amounts. On the basis of the reported products and their amounts, the daily intakes of quercetin, kaempferol, isorhamnetin and myricetin were calculated. Total flavonol intake was calculated in the same manner as described above, i.e., by adding the values for quercetin, kaempferol, isorhamnetin and myricetin.

### 2.2. Validation Procedure

The 24 h recall was taken 3 times (including one weekend day), and the FFQ was administered 2 times to a group of 60 healthy adults aged 20–32 (mean age 21.33 years, SD ± 2.08) at a two-week interval. The healthy population consisted of 48 women (80%) and 12 men (20%). The mean daily intake of selected flavonols was calculated for each participant. Statistical analysis was performed in rStudio software v. 4.2.0. The normality of the distribution of each parameter was checked with the Shapiro–Wilk test.

To evaluate the agreement between the two methods, we compared the mean daily flavonol intakes obtained from the averages of the 3 24 h recalls and the FFQ using the paired-sample *t*-test. Then, to measure the strength and direction of the correlation between the two methods, we computed the crude Pearson correlation for normally distributed variables. The cut-off points used for the correlation coefficient were: <0.20 for low correlation, 0.20–0.49 for moderate correlation and ≥0.50 for high correlation, which was based on other studies of this type [23]. Bland–Altman plots were created to assess and visualize agreement between the two methods [24]. Interpretation criteria indicated good agreement when the difference between the two methods is about one standard deviation, fairly good agreement when the difference between the two methods is about two standard deviations and poor agreement when difference between the two methods is about three standard deviations [25].

Third, for each parameter, subjects were divided into categories relating to the distribution of their dietary intake quartiles. A comparison of the subjects’ categories showed the percentage of participants correctly classified in the same category and the percentage misclassified in the opposite category (opposite quartile). The result permitted an assessment of the proportion of subjects who were classified correctly. A weighted κ statistic was used to account for both the correctly classified percentage and the expected participant proportion classified by chance. The cut-off points used for weighted κ statistics were as follows: <0.20 = low (poor outcome), 0.20–0.50 = moderate (acceptable outcome) and ≥0.50 = high (good outcome).

### 2.3. Reproducibility

Reproducibility between the two FFQ measurements in terms of the mean daily flavonols intake was assessed using the paired-samples *t*-test. For the measurement of the strength and direction of the correlation between the mean daily flavonols and food intakes obtained from two measurements for the same respondent, we calculated the Pearson correlation for normally distributed variables. The cut-off points used for correlation coefficient were as follows: <0.20 for low correlation, 0.20–0.49 for moderate correlation and ≥0.50 for high correlation [23]. For the simplification of the additional analysis, the 140 products were gathered into 12 main categories and 27 subcategories. Then, the mean daily food category and subcategory intakes obtained from the two measurements were analyzed using the paired-samples *t*-test and the Pearson correlation for normally distributed variables for further evaluation of the interpersonal agreement between the two FFQ measurements.

Third, for each parameter, subjects were divided into categories relating to the distribution of their dietary intake quartiles. A comparison of the subjects’ categories showed the percentage of participants correctly classified in the same category and the percentage misclassified in the opposite category (opposite quartile). The results permitted an assessment of the proportion of subjects who were classified correctly. A weighted κ statistic was used to account for both the correctly classified percentage and the expected participant proportion classified by chance. The cut-off points used for weighted κ statistics were as follows: <0.20 for low κ (poor outcome), 0.20–0.50 for moderate κ (acceptable outcome) and ≥0.50 for high κ (good outcome).

### 2.4. Flavonol Intake Assessment in CAD Patients

After the validation of the FFQ in Polish adults, the FFQ was administered to 43 CAD patients (23 men and 20 women) hospitalized between March and August 2022 due to CAD. Inclusion criteria were: (1) CAD diagnosis (2) age ≥ 18 years (3) mental condition that enabled 1-year retrospective dietary interview. On the basis of the FFQ, the mean daily intakes for quercetin, kaempferol, isorhamnetin, myricetin and total flavonols were calculated for each patient. A comparison between CAD patients and healthy respondents was performed using the Mann–Whitney test, and *p* value below 0.05 was considered significant.

### 2.5. Ethical Standards

The study was conducted in line with the directives of the Declaration of Helsinki on Ethical Principles for Medical Research. All participants were informed about the study’s aims, risks and benefits derived from their participation, and in all cases, their informed consent was obtained. The study protocol was approved by the local Bioethics Committee of the Medical University of Lublin (consent no. KE-0254/9/01/2022).

## 3. Results

The comparison of the results from means of 3 24 h recalls and FFQ results (Table 1) indicated that the mean results from FFQ were higher than from 24 h recall (65.72 ± 31.38 mg/day vs. 46.86 ± 38.36 mg/day for total flavonols, 41.82 ± 20.39 mg/day vs. 29.72 ± 17.77 mg/day for quercetin, 13.59 ± 7.37 mg/day vs. 10.82 ± 8.74 mg/day for kaempferol, 5.72 ± 7.65 mg/day vs. 2.83 ± 3.64 mg/day for isorhamnetin and 4.58 ± 2.96 mg/day vs. 3.45 ± 2.59 mg/day for myricetin). The correlations between 24 h recall and FFQ were high for total flavonols (R = 0.50, *p* < 0.001), kaempferol (R = 0.58, *p* < 0.001), isorhamnetin (R = 0.51, *p* < 0.001) and myricetin (R = 0.61, *p* < 0.001) and moderate for quercetin (R = 0.45, *p* < 0.001).

The comparison between 24 h recall and FFQ in terms of quartile classification (Table 2) showed that 46.67% of respondents were classified in the same quartile for total flavonols intake and 5.00% in the opposite quartile. Specifically, for quercetin intake, 43.33% were classified in the same quartile and 6.67% in the opposite quartile; for kaempferol, 50.00% were classified in the same quartile and 1.67% in the opposite one; for isorhamnetin, 43.33% were classified in the same quartile and 5.00% in the opposite one; and for myricetin, 48.33% were classified in the same quartile and 3.33% in the opposite one. The correlations between quartile classification in 24 h recall and FFQ were high for kaempferol (κ = 0.55) and moderate (acceptable) for other categories: total flavonols (κ = 0.41), quercetin (κ = 0.33), isorhamnetin (κ = 0.31) and myricetin (κ = 0.49).

**Figure 2:**
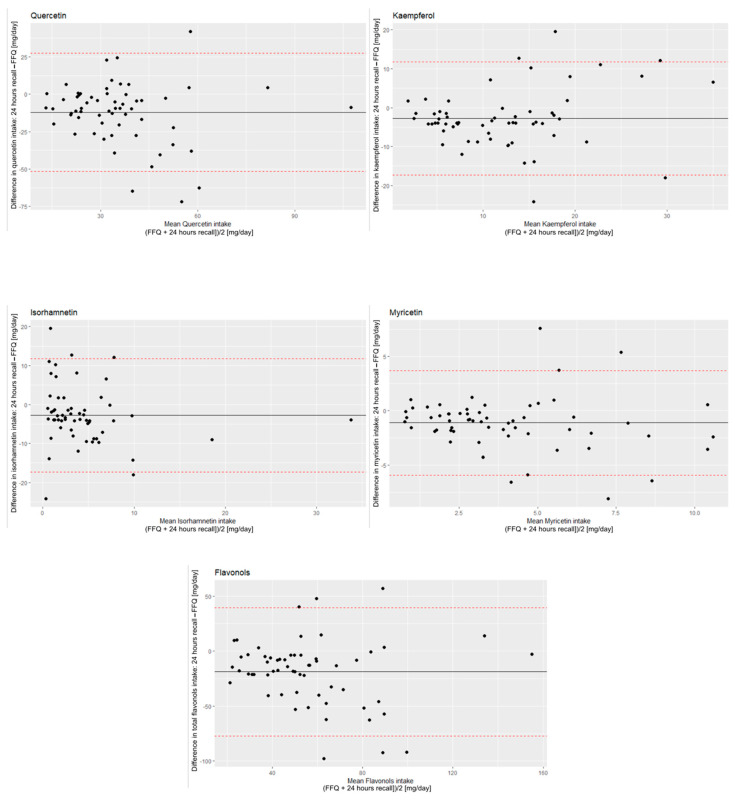
The Bland–Altman plots for quercetin, kaempferol, isorhamnetin, myricetin and total flavonols intake. Continuous lines represent the mean difference between FFQ and 24 h recall result. Dashed line represents 95% limits of agreement.

The Bland–Altman plots (Figure 2) showed that the FFQ provided higher intake estimates for all flavonols compared with the 24 h food records, with relatively wide limits of agreement. The mean differences between the intake assessed in FFQ and 24 h recall were total flavonols, 18.86 (±29.69) mg/day; for quercetin, 12.1 (±20.1) mg/day; for kaempferol, 2.77 (±7.45) mg/day; for isorhamnetin, 2.89 (±6.59) mg/day; and for myricetin, 1.13 (±2.46) mg/day. The Bland–Altman index, which is the percentage of the results classified out of the 95% limit of agreement among all the measurements, was calculated as 6.6% to 8.3%. These results were mainly related to the medium/high flavonol intake. The detailed analysis showed that the results classified as out of the limit of agreement for different flavonols came from the same respondents. Nonetheless, these participants did not present any other distinctive characteristics to predict any subgroup that should be potentially excluded from the application of the FFQ.

The reproducibility assessment for 2 FFQs administered in a two-weeks interval (Table 3) showed high correlations for total flavonols (R = 0.75, *p* < 0.001), quercetin (R = 0.79, *p* < 0.001), kaempferol (R = 0.82, *p* < 0.001) and myricetin (R = 0.85, *p* < 0.001) and moderate for isorhamnetin (R = 0.28, *p* = 0.03). For food main categories and subgroups, high correlations were observed for all main categories (fruits R = 0.65 *p* < 0.001, vegetables R = 0.72 *p* < 0.001, legumes R = 0.91 *p* < 0.001, nuts and seeds R = 0.0.4 *p* < 0.001, sauces R = 0.74 *p* < 0.001, herbs and spices R = 0.59 *p* < 0.001, jam and honey R = 0.87 *p* < 0.001, whole grain products R = 0.89 *p* < 0.001, cocoa R = 0.52 *p* < 0.001, tea and coffee R = 0.93 *p* < 0.001, juices R = 0.76 *p* < 0.001, and alcoholic beverages R = 0.93 *p* < 0.001), and 25 out of 27 subcategories. Among subcategories, moderate correlation was observed only for exotic fruits (R = 0.47, *p* < 0.001) and other leafy vegetables (R = 0.48, *p* < 0.001). Comparisons of quintiles of flavonols and foods intakes by two FFQs were used to assess the reproducibility of the FFQ to classify individuals into the same quartiles (Table 4). Most participants were correctly classified into the same quartile. For flavonols, the lowest was 58.33%, for quercetin, and the highest was 70.00%, for myricetin. Among food main groups and subcategories, the lowest was for solanaceous vegetables at 53.33%, and the highest was for beer, 81.67%.

The correlations between quartile classification in the two FFQ were high for all flavonols (highest for myricetin κ = 0.73 and lowest for quercetin κ = 0.60) and food groups (highest for alcoholic beverages main category κ = 0.82 and beer subcategory κ = 0.82 and lowest for nuts and seeds main category κ = 0.55 and solanaceous vegetables subcategory κ = 0.55.

The comparison of the long-term flavonols intake between CAD patients and healthy respondents revealed significant difference only in isorhamnetin intake (*p* = 0.002). CAD patients consumed significantly lower levels of isorhamnetin than healthy respondents (mean 2.70 ± 2.48 mg/day in CAD patients vs. 5.72 ± 7.65 mg/day in the healthy control). The consumption of other flavonols did not differ significantly between these two groups. For quercetin and total flavonols, intakes were higher in the healthy control (mean 40.84 ± 24.44 mg/day in CAD patients vs. 41.82 ± 20.89 mg/day in healthy volunteers for quercetin and, respectively, 63.40 ± 36.02 mg/day vs. 65.72 ± 31.38 mg/day for total flavonol intake). On the other hand, dietary intakes of kaempferol and myricetin were higher in CAD patients (mean 14.36 ± 8.55 mg/day in CAD patients vs. 13.59 ± 7.37 mg/day in the healthy control for kaempferol, and 5.51 ± 4.17 mg/day vs. 4.58 ± 2.96 mg/day respectively for myricetin). The full data are provided in Table 5.

## 4. Discussion

Numerous FFQs have been developed and validated to assess intakes of different macro- and microelements or other compounds in different populations [3,26,27,28]. They are usually based on general food intake questionnaires and are semi-quantitative [29]. This sort of analysis can lead to omitting some important sources of flavonols, e.g., carob and kale or to the inexact assessment of selected compound intakes due to the semi-quantitative character. Furthermore, there are no validated questionnaires available for isorhamnetin intake assessment. Thus, the particularity of flavonol intake led to the need to design and validate a detailed questionnaire. This is important because other FFQs, even if they count polyphenol intake, do not separate these into selected groups or compounds [20]. Diet is a modifiable risk factor for many chronic diseases, including CAD and cancer, and a valid FFQ could assess dietary intake to reliably relate to such chronic diseases [16,17,30,31]. Therefore, a new FFQ for selected flavonols intake evaluation was created and assessed for validity and reproducibility.

The comparison of the results of mean daily flavonols intake obtained via FFQ and 24 h recall were the basis for the validation of FFQ. The mean results from the FFQ were higher than from 24 h recall. This tendency has been already observed in other studies devoted to FFQ validation, so it was an expected pattern [32,33]. One reason for that is that the FFQ summarized the mean daily flavonols intake throughout a year, which could fluctuate due to the seasonal availability of some products (e.g., fruits and vegetables), while the 24 h recall gathered information during spring, which is a season when the local availability of fruits and vegetables is moderate. Nonetheless, the correlations between the results from FFQ and 24 h recall were significant for total and individual flavonols. The correlations for total flavonols, kaempferol, myricetin and isorhamnetin were high, and for quercetin, it was moderate suggesting that created FFQ is a valid tool for absolute flavonol intake assessment. The Bland–Altman analysis extends the process of validation beyond simple correlation assessment. It tests agreement between a method of interest (FFQ) and a reference method (24 h recall) by plotting the differences between these two dietary methods against the average of the two methods [24]. The mean differences between the intake assessed in FFQ and 24 h recall are presented on a Bland–Altman plot (Figure 2), and they were as follows: for total flavonols, 18.86 mg/day; for quercetin, 12.1 mg/day; for kaempferol, 2.77 mg/day; for isorhamnetin, 2.89 mg/day and for myricetin, 1.13 mg/day. The Bland–Altman index is the percentage of the results classified out of the 95% limit of agreement among all the measurements. The recognized limit for a good agreement between the methods is 5% [34]. This value was exceeded, so the results could not be interpreted as good agreement. However, there are other studies that present tools characterized by Bland–Altman indices within the range of 6–10% and conclude that they can still be effective for epidemiological studies, although not for individual fluctuation assessment [34,35,36,37,38]. Thus, this result was interpreted as a fair agreement between the two methods for epidemiological studies applications.

The analysis of the quartiles of the flavonol intake showed high correlation for kaempferol and moderate correlations for total flavonols, quercetin, myricetin and isorhamnetin. All these results are satisfactory and suggest that the created FFQ can be used for flavonol intake assessment in quartiles.

The analysis of the reproducibility of the FFQ showed that respondents tended to overestimate their flavonol intake more on the first FFQ than on the second FFQ. The mean differences were 13.87 mg/day for total flavonols intake, 8.68 mg/day for quercetin, 2.08 mg/day for kaempferol, 2.16 mg/day for isorhamnetin and 0.96 mg/day for myricetin. However, the reproducibility between the two FFQs was very good. It was high for total flavonols, quercetin, kaempferol and myricetin, and moderate for isorhamnetin. The analysis of the reproducibility between the tested food groups showed high correlations for all 12 main categories (fruits, vegetables, legumes, nuts and seeds, sauces, herbs and spices, jam and honey, wholegrain products, cocoa, tea and coffee, juices and alcoholic beverages) and for 25 out of 27 subcategories. Only the intakes of exotic fruits and leafy vegetables were characterized by moderate correlation, which was still acceptable. The reproducibility analysis of quartile classification showed high correlation for all: total flavonols, individual compounds and all of the main categories and subcategories for food intake. Most of the respondents were correctly classified in the same quartile between the two FFQs. This also indicates good reproducibility of the FFQ in terms of quartile classification.

Flavonols are a group of polyphenols widely distributed in fruits, vegetables and tea. They are broadly investigated in terms of health benefits and the dietary prevention of civilizational diseases. In these circumstances, there has emerged a need for tools for the proper assessment of the relationship between dietary intake of selected flavonols and health-related events. The created FFQ is the response to that need. The FFQ can be used in Polish adult populations for the assessment of absolute values of total flavonols, quercetin, kaempferol, isorhamnetin and myricetin intake, as well as for quartile analyses of their intake.

The dietary intake of flavonols in Polish adult population and CAD patients for total flavonols was higher (65.72 mg/day in FFQ; 46.86 mg/day in 24 h recall and 63.40 mg/day in FFQ respectively) than in the most of the other European populations [13]. The higher intake in 24 h recall was noted only in the UK men (54.88 mg/day in the UK health-conscious population and 51.01 mg/day in general UK population, which could be possibly explained by the high intake of flavonols in this region compared with other European countries including Poland [13]. In the French group from the SU.VI.MAX study, the dietary intake of total flavonols reported on 24 h recall was higher (54 mg/day in men and 50 mg/day for women) than that in the Polish group from the presented study [21]. It is worth to note that the intakes in European populations were calculated only on the basis of 24 h recall and did not analyze the historical dietary intake with dedicated FFQs [13,21].

The proportional intake of quercetin consisted of 63.6% of the total flavonol intake for healthy adults and 64.4% for CAD patients, which was lower than in all the other investigated European populations; for kaempferol, the intakes were 20.7% and 22.6%, respectively, and for isorhamnetin, they were 8.7% and 4.2%, respectively, which were higher than in the other investigated European countries; and for myricetin, the intakes were 7.0% and 8.7%, respectively, higher than in the southern but lower than in the central and northern countries for healthy adults and higher than in the southern and central but lower than in the northern countries for CAD patients [13]. Information about specific flavonols intake from SUVIMAX was not available [21].

Main contributors to flavonols intake in Poland were vegetables (mainly solanaceous and roots and tubers), fruits (mainly exotic fruits and berries), herbs and tea, which matches the trends observed in other European countries [13]. This suggests that dietary choices among Polish adults are probably good in terms of flavonol intake; however, the significant differences between healthy adults and CAD patients were observed only in isorhamnetin intake. The recent studies suggest that flavonols intake or supplementation could potentially reduce blood pressure and other cardiovascular disease risk factors, so the high intake of flavonols in Polish adults could possibly indicate good prognosis [17,19,39]. However, it is worth noting that the studies regarding flavonols supplementation were based on much higher doses of the selected flavonols than occur in the everyday diet. Cardioprotective effects of isorhamnetin were reported only in in vitro and animal model studies [40,41,42]. There have not been any studies regarding isorhamnetin’s role in cardiovascular protection in humans yet.

## 5. Limitations of the Study

Despite the promising results, this study has a few limitations. The 24 h recalls used for validation were gathered within one season, although the choice of this period was determined by the hypothesis that expected intake would comparable with the expected mean intake throughout the year: probably lower than in the summer, higher than in the winter and comparable with autumn, which was based on local availability of the sources of flavonols. The observation period was shortened for better compliance. Future studies should examine the differences between seasons in flavonols intake. Probably the created FFQ could be applied to examine shorter periods of time in the future, but this hypothesis needs further confirmation. Furthermore, the healthy controls were volunteers, and women were more interested in participating in the study than men, which is reflected in the control group structure. The study is also based on questionnaire data acquisition, so it shares all the limitations of this type of methodology. Good parameters of correlation between the two methods (FFQ and 24 h recall) do not alone prove the validity of the FFQ. As the Bland–Altman index exceeded 5%, the full validation of the FFQ failed. Nonetheless, there are available numerous studies in which the tested research method was still considered sufficiently effective even though the Bland–Altman index was within the range of 6–10% [34,35,36,37,38]. This study also has a retrospective design, so prospective analysis of this topic would helpful to confirm the presented results. Nonetheless, this is the first study that linked the intake of isorhamnetin with CAD.

## 6. Conclusions

The results of the conducted analysis showed that intake of total flavonols among Polish adults is higher than in most of the other European countries, which was caused mainly by the high proportional intakes of kaempferol and isorhamnetin. The proportional intakes of quercetin and myricetin in Poland were lower than in the other investigated European countries. The comparison between CAD patients and healthy control revealed significant differences in isorhamnetin intake between these groups. The results suggest that dietary isorhamnetin could have a potential role in CAD prevention.

## Figures and Tables

**Table 1:** Validation FFQ vs. 24 h recall for absolute levels of flavonol intake.

Mean Daily Intake [mg/Day]	Mean from 24 h Recall	SD	Mean from FFQ	SD	95% CI	R	*p*	Paired *t*-Test
Total flavonols	46.86	±38.36	65.72	±31.38	0.285–0.671	0.50	<0.001	<0.001
Quercetin	29.72	±17.77	41.82	±20.39	0.224–0.633	0.45	<0.001	<0.001
Kaempferol	10.82	±8.74	13.59	±7.37	0.388–0.730	0.58	<0.001	0.01
Isorhamnetin	2.83	±3.64	5.72	±7.65	0.292–0.675	0.51	<0.001	0.001
Myricetin	3.45	±2.59	4.58	±2.96	0.428–0.751	0.61	<0.001	0.001

**Table 2:** Validation FFQ vs. 24 h recall for quartiles of flavonol intake.

Mean Daily Intake (24 h vs. FFQ)	Percentage in the Same Quartile	Percent in the Opposite Quartile	κ
Total flavonols	46.67%	5.00%	0.41
Quercetin	43.33%	6.67%	0.33
Kaempferol	50.00%	1.67%	0.55
Isorhamnetin	43.33%	5.00%	0.31
Myricetin	48.33%	3.33%	0.49

**Table 3:** Reproducibility of the FFQ for absolute flavonol [mg/day] and food [portion/day] intakes.

Mean Daily Intake	FFQ1	SD	FFQ2	SD	95 CI	R	*p*	Paired *t*-Test
Total flavonols [mg/day]	72.65	±38.36	58.78	±28.67	0.610–0.842	0.75	<0.001	<0.001
Quercetin [mg/day]	46.16	±24.54	37.48	±18.53	0.668–0.868	0.79	<0.001	<0.001
Kaempferol [mg/day]	14.63	±8.04	12.55	±7.41	0.715–0.889	0.82	<0.001	0.001
Isorhamnetin [mg/day]	6.80	±13.66	4.64	±4.06	0.029–0.500	0.28	0.03	0.21
Myricetin [mg/day]	5.06	±3.34	4.10	±2.80	0.763–0.909	0.85	<0.001	<0.001
Fruits [portion/day]	4.28	±3.86	3.37	±2.44	0.470–0.779	0.65	<0.001	0.02
Citrus fruits [portion/day]	0.78	±0.88	0.57	±0.53	0.466–0.771	0.64	<0.001	0.02
Exotics fruits [portion/day]	1.23	±1.77	0.88	±0.78	0.239–0.643	0.47	<0.001	0.09
Seed fruits [portion/day]	0.52	±0.96	0.40	±0.71	0.818–0.932	0.89	<0.001	0.05
Local fruits [portion/day]	0.58	±0.56	0.56	±0.50	0.658–0.864	0.78	<0.001	0.54
Berries [portion/day]	1.07	±1.31	0.88	±1.05	0.759–0.907	0.85	<0.001	0.04
Dried fruits [portion/day]	0.10	±0.18	0.09	±0.16	0.878–0.955	0.93	<0.001	0.24
Vegetables [portion/day]	6.56	±3.54	5.20	±2.57	0.567–0.822	0.72	<0.001	<0.001
Lettuce [portion/day]	0.81	±1.30	0.66	±0.73	0.583–0.829	0.73	<0.001	0.19
Other leafy vegetables [portion/day]	0.39	±0.86	0.25	±0.42	0.262–0.657	0.48	<0.001	0.17
Solanaceous vegetables [portion/day]	2.20	±1.36	1.75	±1.25	0.672–0.870	0.79	<0.001	0.01
Roots and tubers [portion/day]	1.12	±0.83	0.93	±0.66	0.677–0.872	0.79	<0.001	<0.001
Peppers [portion/day]	0.70	±0.79	0.56	±0.63	0.822–0.933	0.89	<0.001	0.004
Cabbage [portion/day]	0.21	±0.27	0.18	±0.25	0.697–0.881	0.81	<0.001	0.18
Onions [portion/day]	0.99	±0.83	0.78	±0.63	0.670–0.870	0.79	<0.001	0.002
Sprouts [portion/day]	0.13	±0.32	0.10	±0.27	0.408–0.741	0.60	<0.001	0.39
Legumes [portion/day]	0.33	±0.40	0.28	±0.36	0.859–0.948	0.91	<0.001	0.03
Nuts and seeds [portion/day]	0.36	±0.68	0.22	±0.41	0.332–0.698	0.54	<0.001	0.07
Herbs and spices [portion/day]	1.61	±2.04	1.05	±0.99	0.400–0.736	0.59	<0.001	0.01
Sauces [portion/day]	0.40	±0.42	0.37	±0.37	0.605–0.840	0.74	<0.001	0.35
Jam and honey [portion/day]	0.35	±0.40	0.33	±0.41	0.796–0.923	0.87	<0.001	0.57
Jam [portion/day]	0.17	±0.21	0.16	±0.23	0.542–0.810	0.70	<0.001	0.70
Honey [portion/day]	0.17	±0.33	0.17	±0.33	0.939–0.978	0.96	<0.001	0.60
Wholegrain [portion/day]	0.15	±0.21	0.14	±0.20	0.816–0.931	0.89	<0.001	0.22
Cocoa [portion/day]	0.12	±0.17	0.10	±0.14	0.307–0.684	0.52	<0.001	0.42
Tea and coffee [portion/day]	2.56	±1.92	2.22	±1.81	0.880–0.956	0.93	<0.001	<0.001
Tea [portion/day]	1.62	±1.48	1.40	±1.36	0.882–0.956	0.93	<0.001	0.003
Coffee [portion/day]	0.94	±1.10	0.82	±1.00	0.800–0.924	0.88	<0.001	0.09
Other non-alcoholic beverages (juices) [portion/day]	0.60	±0.52	0.56	±0.48	0.633–0.862	0.76	<0.001	0.35
Alcoholic beverages [portion/day]	0.27	±0.44	0.25	±0.38	0.880–0.956	0.93	<0.001	0.37
Wine [portion/day]	0.17	±0.35	0.16	±0.33	0.935–0.976	0.96	<0.001	0.40
Beer [portion/day]	0.09	±0.14	0.08	±0.11	0.652–0.861	0.78	<0.001	0.47

**Table 4:** Reproducibility of the FFQ for quartiles of flavonols and food intakes.

Mean Daily Intake (FFQ)	Percentage in the Same Quartile [%]	Percent in the Opposite Quartile [%]	κ
Total flavonols [mg/day]	63.33	1.67	0.65
Quercetin [mg/day]	58.33	6.67	0.60
Kaempferol [mg/day]	61.67	1.67	0.68
Isorhamnetin [mg/day]	68.33	8.33	0.68
Myricetin [mg/day]	70.00	3.33	0.73
Fruits [portion/day]	66.67	6.67	0.68
Citrus fruits [portion/day]	66.67	8.33	0.67
Exotics fruits [portion/day]	63.33	10.00	0.63
Seed fruits [portion/day]	58.33	1.67	0.64
Local fruits [portion/day]	63.33	5.00	0.66
Berries [portion/day]	60.00	11.67	0.59
Dried fruits [portion/day]	71.67	5.00	0.75
Vegetables [portion/day]	66.67	5.00	0.68
Lettuce [portion/day]	55.00	5.00	0.60
Other leafy vegetables [portion/day]	66.67	5.00	0.68
Solanaceous vegetables [portion/day]	53.33	6.67	0.55
Roots and tubers [portion/day]	55.00	6.67	0.57
Peppers [portion/day]	56.67	5.00	0.62
Cabbage [portion/day]	58.33	10.00	0.58
Onions [portion/day]	55.00	5.00	0.59
Sprouts [portion/day]	65.00	6.67	0.65
Legumes [portion/day]	70.00	1.67	0.76
Nuts and seeds [portion/day]	56.67	10.00	0.55
Herbs and spices [portion/day]	65.00	6.67	0.66
Sauces [portion/day]	66.67	10.00	0.65
Jam and honey [portion/day]	65.00	6.67	0.69
Jam [portion/day]	58.33	5.00	0.63
Honey [portion/day]	61.67	1.67	0.67
Wholegrain [portion/day]	63.33	8.33	0.64
Cocoa [portion/day]	66.67	13.33	0.64
Tea and coffee [portion/day]	70.00	3.33	0.74
Tea [portion/day]	71.67	1.67	0.76
Coffee [portion/day]	78.33	5.00	0.78
Other non-alcoholic beverages (juices) [portion/day]	73.33	5.00	0.73
Alcoholic beverages [portion/day]	76.67	0.00	0.82
Wine [portion/day]	75.00	8.33	0.74
Beer [portion/day]	81.67	5.00	0.82

**Table 5:** Comparison of flavonols intake between CAD patients and healthy control.

Mean Daily Intake [mg/Day]	CAD Patients (n = 43)	SD	Healthy Control (n = 60)	SD	*p*
Total flavonols	63.40	±36.02	65.72	±31.38	0.54
Quercetin	40.84	±24.44	41.82	±20.39	0.53
Kaempferol	14.36	±8.55	13.59	±7.37	0.84
Isorhamnetin	2.70	±2.48	5.72	±7.65	0.002
Myricetin	5.51	±4.17	4.58	±2.96	0.26

## Data Availability

The data that support the findings of this study are available from the corresponding author upon reasonable request.

## Supplementary Materials

The following supporting information can be downloaded at: https://www.mdpi.com/article/10.3390/ijerph191912546/s1, File S1: The list of food groups and products; File S2: Food frequency questionnaire.

## References

[1] Czapla M., Juárez-Vela R., Łokieć K., Wleklik M., Karniej P., Smereka J. (2022). The Association between Nutritional Status and Length of Hospital Stay among Patients with Hypertension. Int. J. Environ. Res. Public Health.

[2] Czapla M., Karniej P., Juárez-Vela R., Łokieć K. (2020). The association between nutritional status and in-hospital mortality among patients with acute coronary syndrome—a result of the retrospective nutritional status heart study (Nshs). Nutrients.

[3] Yue Y., Petimar J., Willett W.C., Smith-Warner S.A., Yuan C., Rosato S., Sampson L., Rosner B., Cassidy A., Rimm E.B. (2020). Dietary flavonoids and flavonoid-rich foods: Validity and reproducibility of FFQ-derived intake estimates. Public Health Nutr..

[4] Galván-Portillo M., Vázquez-Salas R.A., Hernández-Pérez J.G., Blanco-Muñoz J., López-Carrillo L., Torres-Sánchez L. (2022). Dietary flavonoid patterns and prostate cancer: Evidence from a Mexican population-based case-control study. Br. J. Nutr..

[5] Mattioli V., Zanolin M.E., Cazzoletti L., Bono R., Cerveri I., Ferrari M., Pirina P., Garcia-Larsen V. (2020). Dietary flavonoids and respiratory diseases: A population-based multi-case-control study in Italian adults. Public Health Nutr..

[6] Chekalina N., Burmak Y., Petrov Y., Borisova Z., Manusha Y., Kazakov Y., Kaidashev I. (2018). Quercetin reduces the transcriptional activity of NF-kB in stable coronary artery disease. Indian Heart J..

[7] Suri S., Liu X.H., Rayment S., Hughes D.A., Kroon P.A., Needs P.W., Taylor M.A., Tribolo S., Wilson V.G. (2010). Quercetin and its major metabolites selectively modulate cyclic GMP-dependent relaxations and associated tolerance in pig isolated coronary artery. Br. J. Pharmacol..

[8] Wang D., Zhang X., Li D., Hao W., Meng F., Wang B., Han J., Zheng Q. (2017). Kaempferide Protects against Myocardial Ischemia/Reperfusion Injury through Activation of the PI3K/Akt/GSK-3 β Pathway. Mediat. Inflamm..

[9] Lian T.W., Wang L., Lo Y.H., Huang I.J., Wu M.J. (2008). Fisetin, morin and myricetin attenuate CD36 expression and oxLDL uptake in U937-derived macrophages. Biochim. Biophys. Acta—Mol. Cell Biol. Lipids.

[10] Panche A.N., Diwan A.D., Chandra S.R. (2016). Flavonoids: An overview. J. Nutr. Sci..

[11] Zamora-Ros R., Andres-Lacueva C., Lamuela-Raventós R.M., Berenguer T., Jakszyn P., Barricarte A., Ardanaz E., Amiano P., Dorronsoro M., Larrañaga N. (2010). Estimation of Dietary Sources and Flavonoid Intake in a Spanish Adult Population (EPIC-Spain). J. Am. Diet. Assoc..

[12] Sampson L., Rimm E., Hollma P.C.H., de Vries J.H.M., Katan M.B. (2002). Flavonol and Flavone Intakes in US Health Professionals. J. Am. Diet. Assoc..

[13] Zamora-Ros R., Knaze V., Luján-Barroso L., Slimani N., Romieu I., Fedirko V., De Magistris M.S., Ericson U., Amiano P., Trichopoulou A. (2011). Estimated dietary intakes of flavonols, flavanones and flavones in the European Prospective Investigation into Cancer and Nutrition (EPIC) 24 hour dietary recall cohort. Br. J. Nutr..

[14] Cassidy A., O’Reilly É.J., Kay C., Sampson L., Franz M., Forman J., Curhan G., Rimm E.B. (2011). Habitual intake of flavonoid subclasses and incident hypertension in adults. Am. J. Clin. Nutr..

[15] Marniemi J., Alanen E., Impivaara O., Seppänen R., Hakala P., Rajala T., Rönnemaa T. (2005). Dietary and serum vitamins and minerals as predictors of myocardial infarction and stroke in elderly subjects. Nutr. Metab. Cardiovasc. Dis..

[16] Marrero A.D., Quesada A.R., Martínez-Poveda B., Medina M.Á. (2022). Antiangiogenic Phytochemicals Constituent of Diet as Promising Candidates for Chemoprevention of Cancer. Antioxidants.

[17] Popiolek-Kalisz J., Fornal E. (2022). The Impact of Flavonols on Cardiovascular Risk. Nutrients.

[18] Martin M.A., Goya L., Ramos S. (2017). Protective effects of tea, red wine and cocoa in diabetes. Evidences from human studies. Food Chem. Toxicol..

[19] Popiolek-Kalisz J., Fornal E. (2022). The Effects of Quercetin Supplementation on Blood Pressure—Meta-Analysis. Curr. Probl. Cardiol..

[20] Taguchi C., Kishimoto Y., Kondo K. (2021). Validation of Food-Frequency Questionnaires for Polyphenol Intake in Japanese Adults. J. Nutr. Sci. Vitaminol..

[21] Hercberg S., Galan P., Preziosi P., Bertrais S., Mennen L., Malvy D., Roussel A.M., Favier A., Briançon S. (2004). The SU.VI.MAX study: A randomized, placebo-controlled trial of the health effects of antioxidant vitamins and minerals. Arch. Intern. Med..

[22] Bhagwat S., Haytowitz D.B., Holden J.M. (2011). USDA Database for the Flavonoid Content of Selected Foods. Release 3.

[23] Yu A.D., Mumme K.D., Conlon C.A., von Hurst P.R., Gillies N., Heath A.L., Coad J., Beck K.L. (2022). Relative Validity and Reproducibility of a Semi-Quantitative Food Frequency Questionnaire for Determining Nutrient Intake in Older Adults in New Zealand: The REACH Study. Nutrients.

[24] Martin Bland J., Altman D. (1986). Statistical methods for assessing agreement between two methods of clinical measurement. Lancet.

[25] Watson J.F., Collins C.E., Sibbritt D.W., Dibley M.J., Garg M.L. (2009). Reproducibility and comparative validity of a food frequency questionnaire for Australian children and adolescents. Int. J. Behav. Nutr. Phys. Act..

[26] Palacios C., Trak M.A., Betancourt J., Joshipura K., Tucker K.L. (2015). Validation and reproducibility of a semi-quantitative FFQ as a measure of dietary intake in adults from Puerto Rico. Public Health Nutr..

[27] Sadeghi S., Montazeri V., Zamora-Ros R., Biparva P., Sabour S., Pirouzpanah S. (2021). Food frequency questionnaire is a valid assessment tool of quercetin and kaempferol intake in Iranian breast cancer patients according to plasma biomarkers. Nutr. Res..

[28] Somerset S., Papier K. (2014). A Food Frequency Questionnaire Validated for Estimating Dietary Flavonoid Intake in an Australian Population. Nutr. Cancer.

[29] Block G., Hartman A.M., Dresser C.M., Carroll M.D., Gannon J., Gardner L. (1986). A data-based approach to diet questionnaire design and testing. Am. J. Epidemiol..

[30] Christensen K.Y., Naidu A., Parent M.-É., Pintos J., Abrahamowicz M., Siemiatycki J., Koushik A. (2012). The Risk of Lung Cancer Related to Dietary Intake of Flavonoids. Nutr. Cancer.

[31] Woo H.D., Kim J. (2013). Dietary Flavonoid Intake and Smoking-Related Cancer Risk: A Meta-Analysis. PLoS ONE.

[32] Hafizah Y.N., Ang L.C., Yap F., Najwa W.N., Cheah W.L., Ruzita A.T., Jumuddin F.A., Koh D., Lee J.A.C., Essau C.A. (2019). Validity and reliability of a food frequency questionnaire (FFQ) to assess dietary intake of preschool children. Int. J. Environ. Res. Public Health.

[33] Jarvinen R., Seppanen R., Paul K. (1993). Short-Term and Long-Term Reproducibility of Dietary History Interview Data. Int. J. Epidemiol..

[34] Flegal K.M., Graubard B., Ioannidis J.P.A. (2020). Use and reporting of Bland–Altman analyses in studies of self-reported versus measured weight and height. Int. J. Obes..

[35] Pursey K., Burrows T.L., Stanwell P., Collins C.E. (2014). How accurate is web-based self-reported height, weight, and body mass index in young adults. J. Med. Internet Res..

[36] Powell-Young Y.M. (2012). The validity of self-report weight and height as a surrogate method for direct measurement. Appl. Nurs. Res..

[37] Yoong S.L., Carey M.L., D’Este C., Sanson-Fisher R.W. (2013). Agreement between self-reported and measured weight and height collected in general practice patients: A prospective study. BMC Med. Res. Methodol..

[38] Pasalich M., Lee A.H., Burke L., Jancey J., Howat P. (2014). Accuracy of self-reported anthropometric measures in older Australian adults. Australas. J. Ageing.

[39] Menezes R., Rodriguez-Mateos A., Kaltsatou A., González-Sarrías A., Greyling A., Giannaki C., Andres-Lacueva C., Milenkovic D., Gibney E.R., Dumont J. (2017). Impact of flavonols on cardiometabolic biomarkers: A meta-analysis of randomized controlled human trials to explore the role of inter-individual variability. Nutrients.

[40] Huang L., He H., Liu Z., Liu D., Yin D., He M. (2016). Protective Effects of Isorhamnetin on Cardiomyocytes Against Anoxia/Reoxygenation-induced Injury Is Mediated by SIRT1. J. Cardiovasc. Pharmacol..

[41] Zhang N., Pei F., Wei H., Zhang T., Yang C., Ma G., Yang C. (2011). Isorhamnetin protects rat ventricular myocytes from ischemia and reperfusion injury. Exp. Toxicol. Pathol..

[42] Xu Y., Tang C., Tan S., Duan J., Tian H., Yang Y. (2020). Cardioprotective effect of isorhamnetin against myocardial ischemia reperfusion (I/R) injury in isolated rat heart through attenuation of apoptosis. J. Cell. Mol. Med..

