# Supplementary material for: Dietary Isorhamnetin Intake Is Inversely Associated with Coronary Artery Disease Occurrence in Polish Adults
Source: Int J Environ Res Public Health. 2022 Oct 1;19(19):12546. doi: 10.3390/ijerph191912546 (PMC9566513; doi:10.3390/ijerph191912546)
Supplement: Supplementary file 1 [file ijerph-19-12546-s001.zip › Supplementary File S1.pdf]

Categories and subcategories of products

Fruits
Citrus fruits (Lemon, White grapefruit, Red grapefruit, Orange)
Exotics fruits (Pineapple, Banana, Date, Fig, Kiwi, Mango, Melon, Dark grapes, White grapes)
Seed fruits (Cherries, Plum, Peach, Apricot, Nectarine)
Local fruits (Pear, Apple)
Berries (Gooseberry, Chokeberry, White currant, Blueberry, Lingonberry, Black currant, Elderberry, Red currant, Goji berries, Blackberries, Rapsberries, White mulberry, Strawberries, Cranberries - fresh)
Dried fruits (Dried cranberries, Prunes, Raisins)

Vegetables
Lettuce (Iceberg lettuce, Butterhead lettuce, Radicchio lettuce, Chicory, Endive, Watercress, Rocket)
Other leafy vegetables (Dock, Spinach, Beet leaves, Radish leaves)
Solanaceous vegetables (Aubergine, Zucchini, Cucumber, Cherry tomatoes, Tomatoes, Canned tomatoes, Asparagus)
Roots and tubers (Sweet potatoes, Beetroot, Ginger, Turnip, Celeriac, Celery, Fennel, Carrot, Potatoes, Horseradish, Parsnip)
Peppers (Jalapeno pepper, Red pepper, Green pepper, Yellow pepper)
Cabbage (Cabbage, Red cabbage, Sauercraut, Kale, Brussels sprout, Broccoli, Cauliflower)
Onions (White onion, Red onion, Garlic, Leek)
Sprouts (Alfalfa shoots, Radish shoots, Cress)
Legumes (White beans, Red beans, String bean, Peas, Broad beans, Tofu)

Nuts and seeds (Pistacchio, Almonds, Chia seeds)

Herbs and spices (Coriander, Dill, Lovage, Apple vinegar, Oregano, Parsley, Chives, Caper)

Sauces (Ketchup)

Jam and honey
Jam (Cranberry jam, Peach jam, Raspberry jam, Strawberry jam, Plum jam)
Honey (Honey)

Wholegrain (Buckwheat groats, Buckwheat bran)

Cocoa (Cocoa, Hot chocolate powder, Carob)

Tea and coffee
Tea (Black tea, Green tea)
Coffee (Coffee)

Other non-alcoholic beverages (juices) (Elderberry juice concentrate, Lemon juice, Black currant juice, Grapefruit juice, Apple juice, Orange juice, Tomato juice, Multifruit juice, Grape juice, Cherry juice, Pomegranate juice, Cranberry juice)

Alcoholic beverages
Wine (Champagne, Red fruit wine, Red dry wine Cabernet Sauvignon, Red dry wine Shiraz, Red table wine, Sweet dessert wine, White fruit wine, White table wine)
Beer (Beer)
