# Supplementary material for: Dietary Isorhamnetin Intake Is Inversely Associated with Coronary Artery Disease Occurrence in Polish Adults
Source: Int J Environ Res Public Health. 2022 Oct 1;19(19):12546. doi: 10.3390/ijerph191912546 (PMC9566513; doi:10.3390/ijerph191912546)
Supplement: Supplementary file 1 [file ijerph-19-12546-s001.zip › Supplementary File S2.pdf]

FOOD FREQUENCY QUESTIONNAIRE

Product	Portion size [gram]		Never or almost never	1 portion per month	Few portions per month – if so, please give the numer of portions per month?	1 portion per week	Few portions per week – if so, please give the numer of portions per week?	1 portion per day	Few portions per day – if so, please give the numer of portions per day?
**Vegetables and legumes**									
Aubergine	a piece	240							
Sweet potatoes	a piece	230							
Beet leaves	a bunch	280							
Broad beans	a handful	80							
Broccoli	a portion	100							
Brussels sprout	a portion	200							
Beetroot	a piece	105							
White onion	a piece	105							
Red onion	a piece	105							
Zucchini	a piece	600							
Chicory	a piece	70							
Garlic	a clove	5							
Endive	a piece	80							
White beans	a glass	190							
Red beans	a glass	190							
String bean (green)	a portion	200							
String bean (yellow)	a portion	200							
Peas	a spoon	15							
Ginger	a piece	10							
Kale	a handful	20							
Cauliflower	a portion	200							
Turnip	a piece	165							
Caper	a spoon	10							
Cabbage	a glass	110							
Red cabbage	a glass	110							
Sauercraut	a glass	110							
Alfalfa shoots	a spoon	8							
Radish shoots	a spoon	8							
Fennel	a piece	350							
Radish leaves	a handful	15							
Carrot	a piece	45							
Cucumber	a piece	35							
Jalapeno pepper	a piece	4							
Red pepper	a piece	230							
Green pepper	a piece	140							
Yellow pepper	a piece	200							
Parsnip	a piece	170							
Cherry tomatoes	a handful	100							
Tomatoes	a piece	170							
Canned tomatoes	a can	400							
Leek	a piece	50							
Watercress	a handful	45							
Rocket	a handful	20							
Cress	a handful	10							

Iceberg lettuce	a leaf	40							
Butterhead lettuce	a leaf	5							
Radicchio lettuce	a leaf	35							
Celeriac	a slice	60							
Celery	a piece	45							
Dock	a handful	70							
Asparagus	a piece	30							
Spinach	a handful	25							
Tofu	a slice	16							
Horseradish	a teaspoon	10							
Potatoes	a piece	75							
**Fruits and jams**									
Gooseberry	a handful	50							
Pineapple	a slice	80							
Chokeberry	a handful	45							
Banana	a piece	120							
White currant	a handful	60							
Blueberry	a handful	50							
Lingonberry	a handful	50							
Peach	a piece	85							
Lemon	a slice	10							
Black currant	a handful	40							
Elderberry	a portion	100							
Red currant	a handful	45							
Date	a piece	5							
Peach jam	a teaspoon	10							
Raspberry jam	a teaspoon	10							
Strawberry jam	a teaspoon	10							
Fig	a piece	70							
White grapefruit	a piece	260							
Red grapefruit	a piece	260							
Pear	a piece	130							
Apple	a piece	180							
Goji berries	a spoon	8							
Blackberries	a handful	65							
Kiwi	a piece	75							
Rapsberries	a handful	70							
Mango	a piece	280							
Melon	a piece	630							
Apricot	a piece	45							
White mulberry	a handful	30							
Nectarine	a piece	115							
Orange	a piece	240							
Plum jam	a teaspoon	10							
Prunes	a piece	7							
Plum	a piece	20							
Strawberries	a handful	70							
Dark grapes	a handful	70							
White grapes	a handful	70							
Cherries	a handful	70							
Dried cranberries	a spoon	12							

Cranberry jam	a spoon	25							
Cranberries (fresh)	a handful	50							
**Herbs, spices and sauces**									
Honey	a teaspoon	12							
Carob	a spoon	12							
Ketchup	a spoon	20							
Coriander	a handful	1							
Dill	a teaspoon	4							
Lovage	a teaspoon	6							
Almonds	a spoon	15							
Chia seeds	a spoon	10							
Apple vinegar	a spoon	5							
Oregano (dried)	a teaspoon	4							
Oregano (fresh)	a handful	3							
Parsley (fresh)	a teaspoon	6							
Parsley (dried)	a teaspoon	6							
Pistacchio	a spoon	9							
Raisins	a spoon	15							
Chives	a spoon	5							
**Wholegrains**									
Buckwheat groats	a bag	100							
Buckwheat bran	a spoon	8							
**Beverages**									
Black tea	a glass	250							
Green tea	a glass	250							
Coffee	a cup	125							
Cocoa	a spoon	10							
Hot chocolate (powder)	a spoon	10							
Elderberry juice concentrate	a teaspoon	5							
Lemon juice	a spoon	6							
Black currant juice	a glass	250							
Grapefruit juice	a glass	250							
Apple juice	a glass	250							
Orange juice	a glass	250							
Tomato juice	a glass	250							
Multifruit juice	a glass	250							
Grape juice	a glass	250							
Cherry juice	a glass	250							
Pomegranate juice	a glass	250							
Cranberry juice	a glass	250							
Beer	a big glass	500							
Champagne	a glass	100							
Red fruit wine	a glass	150							
Red dry wine (Cabernet Sauvignon)	a glass	150							
Red dry wine (Shiraz)	a glass	150							
Red table wine	a glass	150							
Sweet dessert wine	a glass	150							
White fruit wine	a glass	150							
White table wine	a glass	150							

Do you take any supplements, if so please enumerate them (name, dose)?
